# Editorial: In vivo retinal imaging in animal models

**DOI:** 10.3389/fopht.2023.1309894

**Published:** 2023-10-23

**Authors:** Tae-Hoon Kim, Yannis M. Paulus, Bernhard Baumann, Pengfei Zhang, Shaohua Pi

**Affiliations:** ^1^ Department of Translational Imaging, Genentech, Inc., South San Francisco, CA, United States; ^2^ Department of Ophthalmology and Visual Sciences, University of Michigan, Ann Arbor, MI, United States; ^3^ Department of Biomedical Engineering, University of Michigan, Ann Arbor, MI, United States; ^4^ Center for Medical Physics and Biomedical Engineering, Medical University of Vienna, Vienna, Austria; ^5^ School of Optoelectronic Engineering and Instrumentation Science, Dalian University of Technology, Dalian, China; ^6^ Department of Ophthalmology, University of Pittsburgh, Pittsburgh, PA, United States

**Keywords:** retina, *in vivo* retinal imaging, optical coherence tomography, scanning laser ophthalmoscopy, retina diseases, *in vivo* mouse experiments, ophthalmology

In vivo retinal imaging techniques are invaluable not only because of their non-invasive nature, which allows for longitudinal studies with fewer animals, but also for their precision in tracking disease progression and therapeutic responses across various retinal diseases. Our Special Research Topic, titled “*In vivo retinal imaging in animal models*,” features original research on potential imaging biomarkers for retinal degeneration, the use of in vivo imaging systems in mouse models, and the advancement of retinal imaging systems.

The study led by Bell et al. broadens our understanding of early indicators of photoreceptor damage. This study emphasizes the validation and comparison of imaging biomarkers like retinal infoldings, autofluorescent foci, retinal thickness, and optical coherence tomography (OCT) signal amplitude across three mouse strains—albino BALB/cJ and B6(Cg)-Tyrc-2J/J, and pigmented C57Bl/6J—under two differing vivarium lighting conditions. Elevated light exposure led to photoreceptor changes and inflammation in B6(Cg)-Tyrc-2J/J and BALB/cJ strains. While BALB/cJ showed consistent degeneration, B6(Cg)-Tyrc-2J/J experienced a delay, followed by rapid degeneration. C57Bl/6J showed minimal changes, except in inflammatory infiltrates over a year. Biomarker data, analyzed using Spearman’s correlation and Predictive Power Score matrices, highlighted the photoreceptor outer segments’ signal intensities as a key indicator, especially in BALB/c mice. However, the pigmented B6 mice did not showcase any biomarkers linked to light exposure levels, highlighting the variations in biomarker relevance due to strain specificity. This investigation underscores the importance of acknowledging strain-specific responses as the correlation and predictive values of the biomarkers varied even within strains possessing similar attributes, illuminating the necessity for strain-specific considerations in advancing our understanding of retinal diseases.

The retina provides a window in which to perform high-resolution, quick, inexpensive optical imaging of the central nervous system to better elucidate neurodegenerative diseases such as Alzheimer’s disease (AD). A fascinating article by Batista et al. performed OCT Micron IV imaging of a triple-transgenic mouse model of familial AD (3×Tg-AD) and evaluated OCT biomarkers for early disease detection and diagnosis that could be visualized as early as 1 month of age. Biomarkers that distinguished 3xTg-AD mice versus wild-type mice included total retinal thickness, retinal sublayer thicknesses (primarily inner plexiform layer and inner nuclear layer), and retinal texture. Deep learning neural networks were also performed to facilitate early detection. This important work further lays the foundation that high-resolution optical imaging of the retina coupled with deep learning neural networks can determine biomarkers for diagnosis and early detection of neurodegenerative diseases such as Alzheimer’s disease and that the eye can serve as an important window into the brain health.

Laser-induced photodamage in small animals is one important way of studying retinal pathologies. However, the precision of photocoagulation laser targeting often faces limitations due to manual alignment, and lack of real-time feedback on the location and severity of lesions. In response to these challenges, Rico-Jimenez et al. developed a navigated laser delivery system guided by multimodal retinal imaging specifically tailored for the murine retina. This innovative system combines multimodality OCT and dual-color fluorescence Scanning Laser Ophthalmoscopy (fSLO) which can be used to provide real-time imaging feedback for the laser lesioning module. What sets this system apart is its ability to precisely target both focal and extended area lesions by using three sets of independent scanners for laser lesioning module, fSLO, and OCT, respectively. These three modalities were precisely aligned using grid phantom to ensure accurate co-registration between them. With this device, the authors demonstrated real-time visualization of the lesion formation dynamics and the corresponding changes in retinal morphology using fluorescence-labeled mice. This novel approach brings substantial advantages, enhancing the capacity to visualize the photodamage response and ensuring the utmost precision and repeatability in creating laser-induced lesion models for retinal injury. This represents a significant leap forward in the field, offering researchers a powerful tool to explore and understand retinal pathologies with unparalleled accuracy and real-time guidance.

Tang et al. introduced an advanced retinal imaging system specifically configured for in vivo fundus detection in small animals, integrating SLO and OCT methodologies. This system can generate diverse modality images including SLO reflectance, SLO fluorescein angiography, OCT, and OCT angiography (OCTA), concurrently, overcoming the limitations found in existing systems such as a smaller Field of View (FOV) and susceptibility to sample motion due to slow data acquisition speeds. The proposed system is compact and capable of widefield imaging, offering a FOV of up to 100 degrees and has a rapid OCT A-scan rate of 250 kHz. The system is particularly tailored for small animal eyes, which have smaller pupil sizes and significantly shorter axial lengths compared to human eyes, requiring specialized imaging lenses. The integrated approach allows for the simultaneous generation of images from different modalities, all sharing an identical wide FOV. This system not only provides real-time previews but also significantly enhances the efficiency of acquiring bulk data required for OCTA, improving the quality of angiograms by reducing motion artifacts. The system has been validated through experiments involving the retinas of normal wild-type mice and those modeled with retinal detachment and Choroidal Neovascularization (CNV), proving its reliability in identifying retinal lesions in small animals. This multi-modal system is pivotal for researching various retinal diseases in small animals such as glaucoma, diabetic retinopathy, macular degeneration, etc., providing comprehensive information about the structure, function, and blood flow of the retina, which is essential for further advancements in ophthalmological research. The system’s innovative design and integrated approach notably exceed the capabilities of pre-existing multi-modal retinal imaging systems, especially in terms of the field of view, data acquisition speed, and compactness, addressing the unique challenges associated with small animal retinal imaging.

In summary, this Research Topic of original research articles underscores the recent advancements and applications of preclinical imaging techniques in the animal retina. We believe that this compilation will provide guidance and insights to researchers studying vision impairment in animal models of retinal diseases and assessing retinal structure and function after therapeutic interventions.

## Author contributions

T-HK: Writing – original draft, Writing – review & editing. YP: Writing – original draft, Writing – review & editing. BB: Writing – original draft, Writing – review & editing. PZ: Writing – original draft, Writing – review & editing. SP: Writing – original draft, Writing – review & editing.

